# Debating medicalization of Female Genital Mutilation/Cutting (FGM/C): learning from (policy) experiences across countries

**DOI:** 10.1186/s12978-019-0817-3

**Published:** 2019-11-01

**Authors:** Els Leye, Nina Van Eekert, Simukai Shamu, Tammary Esho, Hazel Barrett, ANSER

**Affiliations:** 1 International Centre for Reproductive Health, Ghent University, Ghent, Belgium; 2 Centre for Population, Family & Health, University of Antwerp, Antwerp, Belgium & International Centre for Reproductive Health, Ghent University, Ghent, Belgium; 3 Health Systems Strengthening Division, Foundation for Professional Development, Pretoria, South Africa and School of Public Health, University of the Witwatersrand, Johannesburg, South Africa; 4 Department of Community and Public Health, Africa Coordinating Centre for the Abandonment of Female Genital Mutilation/Cutting, University of Nairobi, Kenya, Technical University of Kenya, Nairobi, Kenya; 5 Centre for Trust, Peace & Social Relations, Coventry University, Coventry, UK; 6 Academic Network for Sexual and Reproductive Health and Rights Policy, International Centre for Reproductive Health, Ghent University, Ghent, Belgium

**Keywords:** Medicalized FGM/C, Harm reduction, Human rights, Medical ethics

## Abstract

**Background:**

Although Female Genital Mutilation/Cutting (FGM/C) is internationally considered a harmful practice, it is increasingly being medicalized allegedly to reduce its negative health effects, and is thus suggested as a harm reduction strategy in response to these perceived health risks. In many countries where FGM/C is traditionally practiced, the prevalence rates of medicalization are increasing, and in countries of migration, such as the United Kingdom, the United States of America or Sweden, court cases or the repeated issuing of statements in favor of presumed minimal forms of FGM/C to replace more invasive forms, has raised the debate between the medical harm reduction arguments and the human rights approach.

**Main body:**

The purpose of this paper is to discuss the arguments associated with the medicalization of FGM/C, a trend that could undermine the achievement of Sustainable Development Goal 5.3. The paper uses four country case studies, Egypt, Indonesia, Kenya and UK, to discuss the reasons for engaging in medicalized forms of FGM/C, or not, and explores the ongoing public discourse in those countries concerning harm reduction versus human rights, and the contradiction between medical ethics, national criminal justice systems and international conventions. The discussion is structured around four key hotly contested ethical dilemmas. Firstly, that the WHO definition of medicalized FGM/C is too narrow allowing medicalized FGM to be justified by many healthcare professionals as a form of harm reduction which contradicts the medical oath of do no harm. Secondly, that medicalized FGM/C is a human rights abuse with lifelong consequences, no matter who performs it. Thirdly, that health care professionals who perform medicalized FGM/C are sustaining cultural norms that they themselves support and are also gaining financially. Fourthly, the contradiction between protecting traditional cultural rights in legal constitutions versus human rights legislation, which criminalizes FGM/C.

**Conclusion:**

More research needs to be done in order to understand the complexities that are facilitating the medicalization of FGM/C as well as how policy strategies can be strengthened to have a greater de-medicalization impact. Tackling medicalization of FGM/C will accelerate the achievement of the Sustainable Development Goal of ending FGM by 2030.

## Plain English summary

Although Female Genital Mutilation/Cutting (FGM/C) is internationally considered a harmful practice, it is increasingly being medicalized allegedly to reduce its negative health effects, and is thus suggested as a harm reduction strategy in response to these perceived health risks.

The purpose of this paper is to discuss the arguments associated with the medicalization of FGM/C, a trend that could undermine the Sustainable Development Goal (5.3) to end FGM/C by 2030. The paper discusses the reasons for engaging in medicalized forms of FGM/C, or not, by exploring ongoing public discourses in four country case studies: Egypt, Indonesia, Kenya and UK. The discussion is structured around four key hotly contested ethical dilemmas. Firstly, that the WHO definition of medicalized FGM/C is too narrow allowing medicalized FGM to be justified by many healthcare professionals as a form of harm reduction which contradicts the medical oath of do no harm. Secondly, that medicalized FGM/C is a human rights abuse with lifelong consequences, no matter who performs it. Thirdly, that health care professionals who perform medicalized FGM/C are sustaining cultural norms that they themselves support and are also gaining financially. Fourthly, the contradiction between protecting traditional cultural rights in legal constitutions versus human rights legislation, which criminalizes FGM/C.

The paper concludes that more research needs to be done in order to understand the complexities that are facilitating the medicalization of FGM/C as well as how policy strategies can be strengthened to accelerate the achievement of the Sustainable Development Goal of ending FGM by 2030.

## Background

### The trend towards medicalization of FGM/C

The World Health Organization defines the “medicalization” of FGM/C as situations in which FGM/C is practiced by any category of health professionals, whether in a public or a private clinic, at home or elsewhere, at any point in a female’s life (including reinfibulation^1^) [2]. Health professionals involved in medicalization include physicians, assistant physicians, clinical officers, nurses, midwives, trained traditional birth attendants (TBAs), gynecologists/ obstetricians, plastic surgeons, and other personnel providing health care to the population, in both private and public sectors. They may be undergoing medical training, working in the medical sector or be retired [2].

Medicalization of FGM/C continues to rise in many countries despite increasing numbers of countries legislating against the practice. Based on self-reported Demographic and Health Survey (DHS) data in 25 countries, Shell-Duncan and colleagues estimated that 26% of the women in the age cohort 15–49, which equals to nearly 16 million women, report having been cut by a medical professional [3]. Medicalization rates, as the percent of FGM/C performed by a medical professional, are highest in the following five countries: Sudan (67%), Egypt (38%), Guinea (15%), Kenya (15%) and Nigeria (13%), and rates are rising in all of these countries, except Nigeria [3]. The performance of the procedure by skilled medical professionals in any setting is systematically documented through the inclusion of a question on who performs the cutting in the DHS module on FGM/C.

The increasing use of medical staff and equipment has also been noted in Somaliland [4]. Reinfibulation is estimated to affect 20 million women globally and between 10 and 16 million women are likely to experience medicalized reinfibulation. Reinfibulation, medicalized or not, is documented in many countries where infibulation is (highly) prevalent, e.g. in Sudan, Somalia, Djibouti and Eritrea [5] as well as in Europe and North America [6, 7]. This paper will use evidence from four countries (Egypt, Indonesia, Kenya and UK) to explore current debates concerning the medicalization of FGM/C.

### Policies on medicalization of FGM/C

Initially, campaigns against FGM/C stressed the adverse health consequences of the practice, assuming that this would help to raise awareness of the health risks and in turn motivate people to abandon the practice [8]. However, it is speculated that the health approach taken in these campaigns has unintentionally motivated the medicalization of FGM/C, at both demand and supply side [2]. In 2009 the World Health Organisation (WHO), the United Nations Children’s Fund (UNICEF) and the United Nations Populations Fund (UNFPA) condemned the medicalization of FGM/C in any setting [9], however, WHO had already raised this issue 30 years earlier (1979) at an international conference, stating “it is unacceptable to suggest that performing less invasive forms of FGM/C within medical facilities will reduce health complications” [1]. The most recent guidance by WHO on the management of health complications from FGM/C states: “stopping medicalization of FGM/C is an essential component of a holistic, human-rights based approach towards the elimination of the practice” [1].

In December 2012, the United Nations General Assembly adopted the first ever Resolution to ban FGM/C worldwide.[10] Resolution A/RES/67/146 was co-sponsored by two thirds of all UN members and was adopted by consensus of all UN members. Its adoption reflected the universal agreement that FGM/C constitutes a violation of human rights, which all countries of the world should address through ‘all necessary measures, including enacting and enforcing legislations to prohibit FGM/C and to protect women and girls.’ More recently, in September 2015, the global community agreed a new set of development goals, the United Nations Sustainable Development Goals (SDGs), which includes Sustainable Development Goal 5: achieve gender equality and empower all women and girls [11, 12]. This Goal includes a target to eliminate all harmful traditional practices, including FGM/C (SDG 5.3), by 2030, a signal of international political will to end the practice of FGM/C globally.

FGM/C whether traditionally performed or medicalized, is now recognized internationally as a violation of girls’ and women’s rights and as an expression of gendered violence, with a demonstrated impact on women’s sexual and reproductive health. Governments worldwide are thus obliged to take measures to prevent and eliminate FGM/C, including medicalized forms of the practice, and can be held accountable for failing to take steps to prohibit the practice of FGM/C through legislative and other measures. Some countries have increased the prison sentences when health professionals have been convicted of performing FGM/C, and some also provide for the revocation of licenses of health professionals if they perform FGM/C [13]. However, even if the legal framework is put in place, a number of challenges remain. This paper contributes to four current hotly contested debates on the medicalization of FGM/C, namely:

i. That the WHO definition of medicalized FGM/C is too narrow allowing medicalized FGM to be justified by many healthcare professionals as a form of harm reduction which contradicts the medical oath of do no harm.
ii. That medicalized FGM/C is a human rights abuse with life long consequences, no matter who performs it.
iii. That health care professionals who perform medicalized FGM/C are sustaining cultural norms that they themselves support and are also gaining financially.
iv. The contradiction between protecting traditional cultural rights in legal constitutions versus human rights legislation, which criminalizes FGM/C.

## Current debates on medicalization of FGM/C

### When does FGM/C become defined as ‘medicalized FGM/C’ and is medicalized FGM/C an acceptable form of ‘harm reduction’?

Although not specifically addressed in the WHO definition, we argue that medicalization of FGM/C might also include performing less invasive forms of FGM/C, often promoted as ‘a harm reduction strategy’. This form of medicalization has been documented in African countries where FGM/C is prevalent, as well as in European countries and the USA. Indeed, in 2010 the American Academy of Pediatrics issued a position statement in which they suggested that ‘it might be more effective if federal and state laws enabled pediatricians to reach out to families by offering a ritual nick as a possible compromise to avoid greater harm”. Such a nick, or prick, would consist of pricking the prepuce of the clitoris, without removal of tissue. A study in Somaliland, for example, showed that there is a trend towards milder forms of FGM/C, with “pharaonic circumcision” (Type III or infibulation) being replaced by “sunna” cutting [14]. Moreover, the study showed that girls are more likely to undergo the procedure in a medical facility where staff has received at least some medical training. A recent study from Nigeria demonstrated that the campaign and legislation against FGM/C and the training of nurses concerning the health implications of FGM/C made them more cautious and because they knew the complications, they were more likely to only nick the clitoris enough to cause bleeding and thus satisfy parents that the procedure had been done, without removing much tissue [15].

Another complication with defining medicalized FGM/C is whether the use of medical instruments (such as sterile razor blades or surgical blades, forceps), antibiotics and/or anesthetics to carry out FGM/C, especially when used by traditional practitioners, should be considered as a form of medicalized FGM/C. Data on this are notably lacking, and only anecdotal evidence is available. In Guinea, the use of razor blades instead of traditional instruments is attributed to the increasing medicalization of the procedure and sensitization campaigns [16] A qualitative study conducted in four communities in the Nigerian States of Delta, Ekiti, Imo and Kaduna, showed that health workers used a range of essential supplies when carrying out FGM/C: antiseptic, artery forceps, surgical scissors or blades, cotton wool, and antibiotics. They described the steps of the procedure as: “using an antiseptic to clean the area, clamping the tissue with forceps, cutting the tissue with scissors or a surgical blade, applying pressure with cotton wool to control bleeding, cleaning the area again with an antiseptic, and applying an oil or Vaseline”. Some ‘health workers’ mentioned also administering pain relief and prescribing antibiotics [15].

Finally, we want to highlight the issue of medicalized reinfibulation, and how a recent court case in the UK demonstrates the difficulties in defining what constitutes medicalized FGM/C, especially in the context of re-stitching following the birth of a child (reinfibulation). The UK case study (see Table 1) is a demonstration of an unsuccessful legal case brought against a doctor who allegedly performed a reinfibulation and illustrates the difficulty of proving to a court that FGM/C has taken place. However, the huge publicity that occurred during and following the court case made it very clear that medicalized, as well as traditional FGM/C, was against the law and prosecutions would be brought. Since this case in 2015, two further unsuccessful cases have been brought in the UK against two different fathers of girls who have allegedly been subjected to FGM/C. Again, these showed a weakness in the law concerning the testimony of the victims and expert evidence from health professionals who could not agree whether FGM/C had taken place on the girls. However, in February 2019 the first successful case was prosecuted in the UK of a mother who performed FGM/C using traditional techniques, FGM/C on her three-year-old daughter.

**Table 1:** United Kingdom – when does a medical procedure become FGM/C?

In the United Kingdom (UK), FGM/C has been illegal since 1985 when the Prohibition of Female Circumcision Act became law. In 2003 the law was amended under the FGM Act (2003) to include an extra-territorial clause. In 2015 provisions were strengthened under the Serious Crime Act, which extended the scope of extra-territorial offences, granted victims of FGM lifelong anonymity; and introduced a new offence of failing to protect a girl at risk of FGM (Crown Prosecution Service, nd). The law states it is a criminal offence to excise, infibulate or otherwise mutilate the whole or any part of a female’s labia majora, labia minora or clitoris. However no offence is committed by a registered healthcare professional who performs: a surgical operation on a female which is necessary for her physical or mental health; or a surgical operation on a female who is in any stage of labour, or has just given birth, for purposes connected with the labour or birth. It is also an offence to aid, abet, counsel or procure FGM [17]. After 30 years with no prosecutions under the FGM/C legislation, the first prosecution was brought to court in January 2015. It was a high profile case involving an alleged medicalized re-infibulation by a doctor in a Maternity Unit of a National Health Service (NHS) hospital in London. The alleged offence took place in November 2012 when a 24-year-old woman was brought into the Maternity Unit in labour with her first child. It was apparent to the midwife in attendance that she had been subjected to type 3 FGM infibulation (which had been performed on her at the age of 6 in her home country, Somalia) and that this had not been picked up earlier in her pregnancy and was making the birth difficult [18, 19]. The doctor was called in and he cut the woman to facilitate the safe birth of her child [18]. Following the delivery of the child, the doctor, allegedly encouraged by her husband [18], sutured the woman, to stop excessive bleeding. However it was claimed by the prosecution that the suturing exceeded that which was medically required and thus constituted re-infibulation [20]. The case thus revolved around whether a figure of eight suture constituted re-infibulation [19]. In the period between when the alleged FGM took place and the court case, the woman had delivered a second child with no need for surgical intervention and she declined to give evidence in court [19, 21]. The defendants (the doctor and the woman’s husband) were both acquitted of the offence by the jury after only 25 min of deliberation [20, 21]. The case has thrown up a number of issues most notably how re-infibulation is defined, i.e. when does a medical procedure become FGM/C. In this prosecution the case consisted of debates and expert witness evidence concerning one suture (in a figure of eight, part of which involved the stitching of the labia). The prosecution claimed this one stitch constituted FGM. This argument was supported by expert evidence from health professionals including the midwife involved in the delivery room at the time [18]. Other health professionals argued otherwise, stating that as the woman had given birth to a second child without having to be surgically cut then the suturing could not be labeled as reinfibulation. The debate on when a medical procedure crosses the legal line to become FGM has not been resolved. However, whilst the prosecution did not result in a conviction, it did raise the issue of the medicalization of FGM/C in the UK, whether this was inadvertent or deliberate. It is not known if any or how many health professionals are engaged in medicalized FGM/C in the UK, but this highly publicized case provided a stark warning to the medical profession that medicalized FGM will not be tolerated in the UK.	

### Medicalized FGM/C: harm reduction or human rights abuse?

One of the most important reasons given by health care professionals who perform FGM/C is their belief that when it is done by skilled professionals, it reduces the immediate health risks and pain, especially when antiseptic techniques, anesthetic and analgesic medication are used [9]. Health professionals doing FGM/C might indeed be able to control the immediate physical consequences of cutting the genitals, such as the severe pain, bleeding and infections. However, many health professionals who perform FGM/C have limited knowledge of long-term health consequences of the procedure, in particular the mental health implications. Even if women do not report physical after-effects of FGM/C, research suggests that the majority of women subjected to FGM/C have reported mental health problems and emotional disorders with living with the effects of FGM/C [22]. A study by Knipscheer indicated a high level of reporting of severe depression, anxiety and Post Traumatic Stress Disorder (PTSD) by FGM/C survivors [23]. Eisold found that FGM/C can affect the emotional well-being of women throughout their lives [24].

Whilst medicalized FGM/C might minimize – but not avoid - some of the long-term physical consequences of FGM/C, the fact remains that there are no perceived health benefits of the practice itself. It is therefore considered to be against good medical practice and a violation of the medical code of ethics, as even “do less harm” is contradictory to the Oath of Hippocrates ‘do no harm’.

Still, the harm reduction approach dominates the discourse, as is demonstrated by the high numbers and increasing rates of health professionals that engage in performing FGM/C. Health professionals performing FGM/C in order to provide a safer setting for the procedure are ignoring the human rights issues associated with FGM/C, including the right to freedom from violence and discrimination, amongst others. The trend to medicalize FGM/C is worrying, given that its impact on the global campaign and efforts to end FGM/C is still not clear. How the promotion of medicalized ‘safe’ or ‘light’ versions of cutting girls’ and women’s genitals influences these efforts is difficult to assess, but it is commonly believed that promoting medicalized forms of FGM/C communicates the message to practicing communities that FGM/C is acceptable when done by health professionals, and thus is a legitimation of the practice [1]. This harm-reduction approach contrasts with the human rights approach, which states that health professionals performing FGM/C in order to provide a safer setting for the procedure, are ignoring the human rights aspects associated with FGM/C.

Furthermore, the assumption that medicalization reduces harm is not empirically proven. Moreover, in the Indonesian case described in Table 2 there is anecdotal evidence to the contrary, namely that midwives perform more severe forms of FGM/C than traditional practitioners. The case of Indonesia also shows that the government has been oscillating between the human rights approach and the harm reduction strategy. Government policy has played a crucial key role in medicalizing FGM/C in Indonesia, together with strong religious/social norms that underpinned this medicalization.

**Table 2:** Indonesia – is the debate “harm-reduction vs. human rights” meaningful?

Indonesia has one of the highest burdens of FGM/C in the world, with 51% of the girls 0 to 11 years having been circumcised [25]. In Indonesia, it is widely believed that FGM/C is a necessary fulfilment of the Islamic religion [26, 27]. The *fatwa*, a recognised body for the preservation of Muslim culture in a secular-led government, argues in favour of FGM/C, by defining FGM/C as the removal of the membrane that covers the clitoris, through scratching without cutting or incision of the clitoris. FGM/C in Indonesia was traditionally conducted by traditional birth attendants, as well as traditional and religious practitioners. When the government rolled out a maternal health programme to reduce maternal deaths in the 1990s, it transferred duties of maternity care and delivery to midwives. Since then clinics and hospitals have increasingly offered FGM/C as part of the delivery package with midwives being the frequently cited personnel performing FGM/C [28]. The proportion of FGM/C performed by midwives and other medical professionals has sharply increased from 32 to 52% between 2003 and 2013 [25, 29]. Countrywide, two-thirds (65%) and two-fifths (40%) of the FGM/C in urban and rural areas respectively, are now being performed by midwives and other health personnel [25]. The last decade has seen the heightening of the debate on FGM/C in Indonesia leading to periods of banning and unbanning of FGM/C. Activists call for its banning while the *fatwa* religious fathers lobby for its continuation. In the 1990s and early 2000s the government was silent on the WHO’s global call to eliminate FGM/C [30]. In the absence of government policy and intervention, midwives responded to women’s demands for FGM/C by conducting FGM/C in health facilities to those who requested it [28]. Following the call for the respect for women and girls’ rights surrounding FGM/C, the government banned FGM/C in 2006. In response, the *fatwa* lobbied the government to rescind the ban arguing that FGM/C was a cultural rite of passage for all Islamic women and must be provided upon parental request on behalf of their children, but that it must be done without causing psychological or physical danger to the woman or girl. Instead of maintaining its stance on the ban, the government gave in and spelt out conditions under which FGM/C could be done. A standard operating procedure allowing only medical personnel to conduct FGM/C in a safe and hygienic manner and to children of parents who requested it was then put in place. In 2014 women’s organizations successfully contested the policy arguing that FGM/C has no medical benefits for women and girls as opposed to male circumcision. Despite the ban that prohibits FGM/C being in place, no sanctions are given for those who transgress this law. Women’s organizations recommended that the government should address the problem, including providing rehabilitation to women living with FGM/C, criminalise the practice and campaign against the practice [25]. Medicalized FGM/C is argued to be a better of the two evils (medicalized versus traditional FGM/C) in that it is done by trained and skilled health professionals in hygienic and medically controlled situations compared to the traditional birth attendants who conduct it in uncontrolled settings with severe pain and complications [26]. However, the opposite has been reported as midwives in Indonesia, were found to perform more invasive and painful forms of excision in 68–88% observed cases compared to 43–67% cases by traditional providers [29]. There is also some concern that as FGM/C has become more medicalised, more physically invasive forms of FGM/C are now more common. However, there is also some evidence that midwives who disagree with FGM/C are performing type 1 or type 4, to satisfy parents that FGM/C has taken place but at the same time minimizing the risk to girls, thus demonstrating that the human rights arguments might gain some impact on the medicalization of FGM/C in Indonesia. In a few instances midwives are said to provide “psychological FGM/C” and not real FGM/C. Hidayana et al. argue that since parents do not know how the midwives conduct FGM/C, some midwives who do not support it pretend to be doing FGM/C to fulfil the client’s request [28]. This case also demonstrates that medical professionals are impacted on by the same social norms as parents and stresses the role medical professionals play in ending FGM/C, which is further discussed in the following section.	

### Medicalized FGM/C: reflecting the social norm or used to justify financial gain?

One aspect that plays a key role in health care professionals deciding to do FGM/C is that they commonly share the same social norms regarding cutting the genitals of girls and women, hence resisting the pressure or the demand to do FGM/C from the community is challenging. A study from Nigeria for example, demonstrated that most health workers that engage in FGM/C do so because they share the same FGM/C beliefs as the community they serve, and this was evidenced by the fact that four out of five health workers with daughters had also cut their own daughters [15]. Another study, from Sudan, concluded that medicalization is primarily driven by the demand motivated by social norms [31].

The patriarchal nature of FGM/C underpins many of the arguments to continue FGM/C, whether it is medicalized or not, and parallels between FGM/C, patriarchy and female genital surgeries have been discussed elsewhere by various scholars (see for example Pedwell C [32], Ogbe E et al. [33]).

However, the financial gains to perform FGM/C for both health professionals and parents should not be underestimated, as FGM/C can bring in additional income to health professionals and for parents it can mean a higher bride price/dowry can be expected when their daughter is married. Health professionals’ motivation to perform FGM/C is reinforced by the fact that many health systems in countries where FGM/C is prevalent are weak, and so extra financial income is attractive. Serour suggests that medicalization of FGM/C is a major source of income for those who perform it. Fees are high, especially in countries where FGM/C is illegal [9, 34].

This is demonstrated by the case study that looks at Egypt, where medical doctors have taken the lead in the medicalization of FGM/C, often arguing that as FGM/C is a strong social norm and will happen whatever, that it is better that it is performed by a medical doctor than a traditional practitioner (Table 3). It has also been argued that many of these doctors support the practice for cultural and religious reasons and in addition make a good livelihood from performing the procedure. Despite cases where girls have died following medicalized FGM/C, few successful prosecutions have taken place against a medical professional in Egypt [42]; a country where medicalized FGM/C is highly prevalent and numbers rising. The Egyptian case study shows us the importance of the context in which FGM/C arises.

**Table 3:** Egypt – the contradiction between social norms and legal frameworks

Worldwide more than half of all medicalized FGM/C procedures are performed in Egypt [3] and medicalization rates in Egypt are rising. Available data show that the percentage of girls cut by health professionals increased from 55% in 1995 to 77% in 2008 [35] and 84% in 2014 (EDHS, 2014). In Egypt, policies and laws related to the medicalization of FGM/C have undergone a number of shifts. In 1994, in an attempt to improve the safety of FGM/C, the government gave its consent for FGM/C when performed by health personnel in public hospitals [35]. This government consent aimed at improving the safety of FGM/C, in a context where people viewed the practice as inevitable. The Egyptian Minister of Health at the time stated that the medicalization of FGM/C would reduce complications and eventually end the practice [9, 36]. Women’s rights and health advocates criticized the consent as government endorsement of FGM/C. In 1995, after the death of a girl in a hospital during a FGM/C procedure [38], this policy was revised. First governmental hospitals, and later private hospitals were banned from performing the procedure, except ‘when medically necessary’. The prerequisite of medical necessity functioned as a loophole until 2006 [35, 36, 38]. In 2007 further restrictions banned all state-licensed health workers in either government or private clinics from performing FGM/C. In 2008 performing FGM/C was criminalized in the penal code [35]. Initially FGM/C was covered as a misdemeanor, imposing the penalty of imprisonment between 3 months and 2 years on practitioners of FGM/C. In 2016, following several deaths of girls while undergoing FGM/C, the law was strengthened and enforced with increased sentences. In 2016 the penalty of imprisonment was raised from five to 7 years for medical practitioners. If the practice led to death or permanent disability the imprisonment could be up to 15 years. Moreover, a penalty of imprisonment between one and 3 years was imposed for any individual who escorted the victims of such crimes to the perpetrators [39]. However, a number of recent studies reveal that despite these policies and legal restrictions the medicalization of FGM/C continues in Egypt [35, 40, 41].	

### FGM/C: cultural rights versus human rights?

Both the Egyptian case discussed above and the Kenyan case discussed hereafter (Table 4) demonstrate how the law has limited influence in contradiction with culture and tradition. It shows how FGM/C is embedded in cultural and traditional norms and rights that are considered by proponents to prevail over the law of the country.

**Table 4:** Kenya – the intersectionality between tradition, culture and human rights

Kenya witnessed a gradual decline in prevalence of FGM/C from 38% in 1998 to 21% in 2014 (KDHS 2014). However, over the same time the rates of medicalization have been on the rise, increasing from 34% in 1998 to 41% in 2008–09, followed by a subsequent drop in 2014 [3]. More worrying are the rates of medicalization of FGM/C of girls as facilitated by their parents in Kenya which is reported to be 20% [40]. Kenya has taken steps towards criminalizing FGM/C, which is evidenced by the various policies and laws passed in the recent past such as the Prohibition of Female Genital Mutilation Act 2011 and the Child Act policy. However, there are a number of challenges that Kenya has faced with regard to the implementation of these laws and policies. For example, on 13th June 2014, after some perpetrators of FGM/C were arrested [43], more than 500 agitated women from the Maasai community held a protest at a shopping center in Kajiado Central, advocating for FGM/C and calling for the government to allow them to practice FGM/C. The women demonstrated against the enforcement of the law in their county after the arrest of the parents of a 13 year-old girl who died in a botched traditional FGM/C procedure. The protesters cited the Kenyan Constitution, which provides for the protection of cultural and traditional rights and subsequently their right to practice FGM/C viewed as an age-old custom that is believed to be necessary for womanhood. Such cases begin to raise concerns about the need to sensitize and create awareness among members of communities that practice FGM/C about harmful practices and how they infringe on girls and women’s rights to bodily and mental integrity. Communities need to de- and re-construct social norms in order to make progress in achieving the SDGs. One of the latest challenges that has captured both local and international attention is the recent court case, filed by a Kenyan, female medical doctor petitioning the High Court to overturn the law that outlaws FGM/C in Kenya. The medical doctor argued against the term ‘mutilation’ which she viewed as a ‘misnomer’, and reiterated “female circumcision was part and parcel of African cultural practices before colonialism, and as such should not be made illegal”. She added, “[ …] once you reach adulthood there is no reason why you should not make that decision”. She argued that “legalizing female circumcision will make it easy for those who want to undergo it to seek the best medical services, thus making the procedure safe” [44]. In her discussion, she also justified the medicalization of the practice by saying that “female circumcision [ …] can be made safe arguing that it is a minor surgical procedure that does not require anesthesia or being put into a theater” [44]. It demonstrates that FGM/C is a practice based on cultural beliefs and deeply embedded social norms; even well educated health care providers find it hard to not comply with the prevailing social norms.	

As alluded to in the Kenyan case of a medical doctor supporting the medicalization of FGM/C, there may be gaps in the law that proponents of FGM/C might use to push their agenda. This case indicates that some medical practitioners themselves do not only medicalize, or support it, but do so by exploiting gaps in the judicial system hence derailing progress made towards abandonment of FGM/C.

## Discussion

Tackling the medicalization of FGM/C needs to consider the contested issues surrounding the debate of medicalized FGM/C. In the current paper we discussed four important issues and dilemmas that should be taken into account: the trouble with defining FGM/C, the need to contextualize FGM/C, the debate of harm-reduction versus social norm and the difficulty of applying a law when it contradicts cultural values and social norms. In conclusion to this paper we want to translate the discussions above to some suggestions for the way forward.

### Policy emphasizing the human rights approach

As demonstrated there is a tension between a pragmatic harm reduction approach maintained by some health professionals and the human rights approach that seeks to safeguard girls and women’s bodily integrity. Social and religious norms supporting the practice of FGM/C pose serious challenges to the implementation of legislation that aims to protect the human rights of women and girls.

In both Egypt and Indonesia the governments have at various times supported the medicalization of FGM/C as a harm reduction strategy, often under great pressure from religious leaders, resulting in a confused response to FGM/C and its medicalization which undermined efforts to end the practice in line with international agreements. In Kenya, Egypt and Indonesia, FGM/C practicing communities and the health profession have been very vocal and at times militant in advocating against national legislation banning FGM/C. Very often these groups have used constitutional arguments such as the preservation of cultural and traditional rights, to support their case. These three case studies reveal that at various times over the last 20 years the harm reduction approach to FGM/C has taken policy precedence over the human rights approach to FGM/C.

FGM/C is a strong social norm that makes it difficult for individuals to challenge, as the practice often occurs in societies where norms of collectivity are predominant. The impact of these different settings on social norm change and human rights is not in the scope of this paper and has been discussed elsewhere by various authors (see for example Diabate et al. [45], UNICEF [46], Leye et al. [47]).

There is now a growing momentum in many high FGM/C prevalence countries and others, to tackle FGM/C from a human rights perspective, 25 years after the World Conference on Human Rights held in Vienna, Austria in 1993 accepted that FGM/C was a violation of human rights. In 2008 the United Nations Special Report on Torture stated that violence against women, including FGM/C can be considered a violation of the Convention Against Torture.[51] Regionally several treaties and consensus documents call for the protection of the rights of women and girls through the abandonment of FGM/C. These include the African Charter on Human and People’s Rights (The Banjul Charter) and the Protocol on the Rights of Women in Africa (Maputo Protocol), the African Charter on the Rights and Welfare of the Child, and the European Convention for the Protection of Human Rights and Fundamental Freedoms [30]. More recently, in September 2017 at a meeting in Egypt jointly organized by UNFPA and the League of Arab States, public statements were made by National Doctors’ Syndicates and Medical Councils as well as the National Midwives Associations in the Arab Region to end the medicalization of FGM/C.

### Educating health professionals on FGM/C and its consequences

Awareness raising on the sexual and reproductive health consequences of FGM/C and the human rights violations, as well as building capacities through inclusion of FGM/C in curricula or postgraduate training of professionals likely to deal with FGM/C are some of the most commonly used strategies to involve health professionals in countering medicalization. From the case studies discussed above the importance of having a clear definition of medicalized FGM/C, such that it’s clear to health professionals when they are performing FGM/C, and thus breaking the law, is highlighted. Moreover, they should be aware of the negative psychological and physical consequences of performing FGM/C.

Training of health care professionals on FGM/C can vary across and between countries and can take different forms, such as the provision of specific training workshops on medicalization or general training on FGM/C, the inclusion of FGM/C in medical curricula, or the development of e-learning modules or other tools on FGM/C. It should be noted however, that very few of these training and capacity building efforts, especially regarding medicalization, are evaluated, hence it remains unclear what the most effective methods of awareness raising amongst health professionals might be. Moreover, a recent analysis of the evidence on knowledge, experiences and attitudes of health professionals towards FGM/C showed that there are six areas for improvement for health care providers. These areas are: knowledge of FGM/C and its consequences, adherence to FGM/C protocols and guidelines, socially constructed acceptance of FGM/C, knowledge of legislation and legal status of FGM/C, condoning, sanctioning or supporting FGM/C and information and training to work with women and girls living with FGM/C [48]. This list indicates that much work still needs to be done.

It is commonly assumed that the reproductive and sexual health consequences, the legal repercussions as well as the human rights dimension should be part of any FGM/C module in the curricula of health professionals. The WHO Guidelines (2016) on the management of health complications from FGM/C, are useful for designing pre- and in-service professional training curricula for health care providers, and include the above-mentioned aspects. However, too often, FGM/C is not included in curricula on a systematic basis, and/or medicalization and the preventive role of health professional is not addressed at all. Moreover, capacity-building on how to resist pressures from the community, as well as communication techniques for social norm change are rare. A study from Nigeria showed that health workers should be educated and empowered to advocate for the abandonment among patients but also among fellow health workers [15]. Studies from The Gambia showed that training programmes should be modeled to fit the specific characteristics of the trainees in terms of sex and ethnicity [49].

### Detangling professional norms from social norms

The above demonstrates that any effort to deal with medicalized FGM/C should take into account the context in which it occurs. Health care providers’ understanding about FGM/C and how their opinions are shaped by social norms should be unpacked. Many health professionals are not aware of the long-term health implications of FGM/C and the fact that it is a violation of human rights and a breach of medical ethics, despite many regional and global protocols cited above condemning it. Moreover, health professionals often share the social norms of FGM/C being an important cultural tradition. Additionally, the financial reward for performing FGM/C is attractive to health professionals, especially in a weak health system.

We are therefore advocating that health professionals receive training to raise their knowledge of the issues surrounding FGM/C and the awareness that performing FGM/C is in contradiction with the Oath of Hippocrates ‘you should do not harm’. In particular, medicalization of FGM/C and how to tackle it should be part of any curriculum of health professionals (pre and postgraduate training). The legal interpretations of what constitutes a crime with regards to medicalization of FGM/C need to be made clear among health professionals.

Codes of conduct or position statements by professional organizations have been issued both in Western countries as well as in countries where FGM/C is most prevalent. Some of these position statements have caused controversy, such as the 2010 Statement by the American Association of Pediatrics that promoted the performance of a ‘ritual nick’. This statement was revised after outrage and fierce opposition by WHO and others. The European Academy of Pediatricians on the other hand, clearly states: “It also calls upon all physicians to help to stop this practice. The practice of offering a “clitoral nick”, a minimal pinprick, must also be condemned as an unnecessary and extremely painful procedure [50]”.

### Motivate health care providers as agents of change for ending FGM/C

Even though health professionals are at the core of the medicalization issue, they can and are targeted as part of the solution to reverse the medicalization of FGM/C. Given that they are important role models in societies, they are often key in becoming agents of change regarding FGM/C. However, a scoping survey would need to be conducted in each country where medicalized FGM/C is performed to assess the knowledge, attitudes and practices of health professionals in the practicing of medicalized FGM/C.

From our discussion above, it is clear that more attention should go to how health care professionals can be used as agents of change for ending FGM/C. This can be done through: 

- Including, more systematically, the human rights framework and the ethics of medicalization of FGM/C in curricula of health professionals’ education and training.
- Building bridges between sectors: linkages between health professionals and legal stakeholders should be explored and reinforced in order to make the implementation of laws banning FGM/C more effective.
- Establishing collaborations between health professionals and religious leaders to agree that FGM/C is not a religious requirement and to communicate this to FGM/C practicing communities.
- Developing strategies on how health professionals can deal with social pressures from the community wanting to continue with FGM/C and to challenge the social norms perpetuating the practice.
- Urging Professional Medical Associations to reinforce the unethical nature of the medicalization of FGM/C and produce public statements and protocols advocating for the ending of FGM/C whether performed in traditional or medicalized settings, including reinfibulation.

## Conclusion

This paper has discussed the complex ethical debates that accompany the medicalization of FGM/C, and the contradictions between the social and cultural norms supporting the continuation of FGM/C and the human rights of women and girls. It is clear that more attention should go to how health care professionals can be used as agents of change for ending FGM/C. It is also clear that more research needs to be done in order to decipher the code that will facilitate the detangling of these social norms from health professional norms and human rights. It is essential that we have a deeper understanding of the issue and the process of medicalization of FGM/C if the United Nations SDG 5.3 of ending FGM by 2030 is to be achieved.

## Data Availability

Not applicable

## Abbreviations

ANSER: Academic Network for Sexual and Reproductive Health and Rights Policy
DHS: Demographic and health survey
EDHS: Egyptian Demographic and Health Survey
FGM: Female genital mutilation
FGM/C: Female genital mutilation/cutting
KDHS: Kenyan Demographic and Health Surve
NHS: National Health Service
PTSD: Post traumatic stress disorder
SDG: Sustainable development goal
TBA: Traditional birth attendant
UK: United Kingdom
UNFPA: United Nations Populations Fund
UNICEF: United Nations Children’s Fund
WHO: World Health Organization

## Footnotes

1 Reinfibulation is the procedure to narrow the vaginal opening in a woman after she has been deinfibulated (i.e. after childbirth); also known as re-suturing [1]
